# Sleep, Scent, and Household Medical Care in Early Modern England

**DOI:** 10.1093/hwj/dbag002

**Published:** 2026-03-25

**Authors:** Holly Fletcher, Sasha Handley

**Affiliations:** University College London; University of Manchester

**Keywords:** Sleep, Health, Material Renaissance, Domestic Medicine, Senses, Smell, Early Modern

## Abstract

Sleep loss was a vital health concern for early modern people, and inducing sleep was essential to healthcare practices. This article traces how English householders sought to procure sleep. Examining the botanical materials, methods and knowledge underpinning soporific recipes in manuscript and printed collections c.1500–1750, we identify olfactory manipulation as the principal strategy in homemade sleep therapies. Scent-based remedies were developed domestically through material experimentation, drawing on the expanding range of soporifics resulting from global trade and colonialism. These experiments are an important facet of the ‘material Renaissance’, which became embedded in the gendered labour regimens of household life.

Take anniseeds, bruise them and boyle them in a little red rose water, then put them in a little bag of the bigness & length of your little finger, sew a string to each end of the bag, and tye it upon your upper lip, just under your nose.^1^

This scent-based sleep therapy designed ‘For One that Cannot Sleep’ sits in a manuscript recipe book compiled by several generations of the Orrery family, who were related by marriage to the Anglo-Irish natural philosopher Robert Boyle (1627–1691).^2^ Boyle famously analysed the soporific qualities of poppy seeds in his natural philosophical work, but the sleep therapies in this manuscript (Wellcome MS 1340) offer a wider view of the experimental sleep care practices that permeated household medical practice in early modern England, and of the people engaged in them.^3^ In this article we draw on this manuscript and many others like it to explore how middling and elite women and men, often with the assistance of domestic servants, experimented with a growing array of sleep recipes to exert agency over their bodies and healthcare regimes and to generate new knowledge about matter and material processes.

Michelle DiMeo’s careful analysis has identified the hand of Margaret Boyle, Countess of Orrery (1623–1689), as the original compiler of Wellcome MS 1340. Margaret was wife to Roger Boyle (1621–1679) and sister-in-law to Robert, and her surviving correspondence shows that she played a leading role in caring for the health of her immediate family and wider kin.^4^ Wellcome MS 1340 was not the work of a single author, however, and a later hand (possibly one of Margaret’s daughters) expanded and ordered the recipe collection, which also features multiple recipe attributions to a wide circle of female and male family members and friends. The manuscript was thus a collaborative and multi-authored venture with a powerful female knowledge base, and also one whose compilers displayed a particular interest in sleep therapies. It contains nine separate recipes to procure sleep – in the instance cited here, by allowing the ‘cold smell’ of crushed aniseeds and rosewater to infuse the patient’s brain, quieten the animal spirits that resided there, and accelerate sleep’s onset.^5^ Sleep therapies of this kind, we argue, formed a key area of domestic medical experimentation because of the vital role that sleep played in early modern health care, and because of the increased capacity for therapeutic intervention with the many different soporific substances, smells, and material devices afforded by the ‘material Renaissance’.

Temperate sleep was valued as an end in itself in early modern health care. The Elizabethan physician and schoolmaster Thomas Cogan (1545?–1607) captured its importance when he declared that ‘The Benefit of sleepe, or the necessity rather needeth no proofe, for that without it no living creature may long endure, according to that saying of the Poet *Ovid*’.^6^ Cogan’s statement featured in his popular healthcare guide *The haven of health*, first published in 1584, in which he drew a direct relationship between the long-term health and happiness of human bodies and habitual patterns of temperate sleep. Cogan’s valuation of sleep was widely shared. ‘Sleeping and Watching’ (waking) formed one of the ‘Six Non Natural Things’ – a set of principles for healthy living designed to guide bodily activity and medical care.^7^ In addition to sleep these included air, food and drink, motion and rest, evacuation and retention, and the passions or emotions. The non-natural categories frequently structured medical regimen guides in the sixteenth and seventeenth centuries, which were exceptionally popular in England’s growing ‘medical marketplace’.^8^ Inducing sleep in a safe and effective manner was thus essential to the practice of health care.

Examining over a hundred collections of household medical recipes from c.1500–c.1700, this article identifies an experimental culture of sleep-therapy production, alongside the material and medical expertise that underpinned it. Soporific ingredients were carefully sourced and selected by female and male householders, their properties analysed, and recipes customized to treat particular illnesses. Recipes were also adjusted to account for differences of age, bodily constitution, and humoral complexion, and to cater for different purses. Perfecting such recipes for the needs of particular patients relied on an extensive process of trial and error, which was key to domestic medical experimentation.

Practices of medical care within early modern households have been well documented, but there remains a shortage of detailed case studies illustrating how such practices met the essential health care needs of daily life.^9^ Our findings address this lacuna by drawing a direct connection between material experiment and sleep therapeutics, and by significantly expanding the assortment of medical uses to which soporific remedies were put in the service of domestic health care. The sleep recipes we analyse addressed periodic episodes of sleep loss, but they were also used to treat more commonplace ailments such as fevers, headaches, joint pain, and congestion, which regularly afflicted women, men, and children.

Our evidence also broadens the spectrum of botanical ingredients that were incorporated into home-made sleep therapies. The early modern world of botanical soporifics extended far beyond the poppy-based remedies that historians have generally prioritized.^10^ This expansion, which extended opportunities for therapeutic intervention, was due in part to a growing familiarity with the pharmacological properties of African, American and Asian soporific plant species that followed in the wake of exploration, colonialism, and global trade.^11^

The scale and complexity of domestic experiment with soporific therapies that we uncover represents a fresh chapter in the early modern ‘material Renaissance’ – a cultural and intellectual movement that valorized material experiment and sensory experience as superior epistemological practices.^12^ Embodied cultures of making and material transformation have been established as central to early modern fashion, craft, and artisanal innovations in the scholarship of Ulinka Rublack, Pamela H. Smith, Stefan Hanß, and others.^13^ The singular importance of recipes to practices of material and scientific experiment is also widely recognized by early modern scholars, and a body of work published in the last couple of decades illustrates a world in which men and women from many walks of life were deeply engaged in the observation, evaluation, and reworking of natural materials, whether animal, mineral or vegetable for a variety of ends.^14^ This remarkable culture also responded to concerns for improved health and wellbeing. Trials and tests were widely undertaken to evaluate the properties and effects of different *materia medica* by physicians at princely courts, in hospitals and universities, and by a range of unlicensed healers and lay practitioners.^15^ Practices of material observation, assessment, and adjustment were especially marked in relation to fabled panaceas and drugs reputed to cure potentially fatal diseases such as plague.^16^

Much less attention has been paid to the modes of experimental thinking and therapeutic trials that were dedicated to more everyday aspects of domestic health care, and that foreground their makers as energetic medical practitioners and environmental agents.^17^ This article therefore aims to establish the transformative impact of the material Renaissance on the therapeutics of sleep care among lay practitioners of medicine. The first impact we trace is the burgeoning repertoire of soporific plants that were used in domestic sleep therapies. This range of ingredients overlapped with the soporific pharmacopoeia acknowledged by the most influential herbalists of the day, but it also diverged from them in significant ways, revealing a semi-independent culture of therapeutic experiment and medical knowledge-making among female and male householders. The second impact identified here is the developing sophistication of a distinctive therapeutic method for procuring sleep, namely olfactory manipulation. Pinpointing olfactory manipulation as the principal mode of operation within homemade sleep therapies, we trace the emergence of a flourishing category of scent-based sleep remedies by the early eighteenth century. The increased number of such therapies within recipe collections rested on their capacity to cool and soothe the brain and quiet the animal spirits. The brain was acknowledged as the main organ of olfaction and as the seat of the animal spirits, whose motion had a unique power to affect sleep patterns. The cultural and medical association between sleep and the brain was pronounced in Aristotelian medicine, but it reached new heights in the seventeenth and early eighteenth centuries as new anatomical experiments identified distinct brain regions as the chief regulators of human sleep.^18^

The turn to scent-based home-made soporific remedies over the course of the early modern period strongly suggests that many lay medical practitioners were aware of the latest advances in sleep physiology, and were actively engaged in therapeutic experiments designed to optimize sleep care. The first duty of a housewife, and indeed a patriarch, was to safeguard family health, and sleep loss was clearly a vital health concern. In this article we begin with an examination of the practice of recipe collecting in early modern England, and go on to analyse the expanding range of botanical soporifics and therapeutic knowledge relating to sleep in contemporary herbals and recipe collections. Finally we focus on sleep-inducing recipes, in manuscript and print, designed for direct bodily application and for scenting bedding textiles and sleeping chambers. Women’s especially prominent role in developing the latter shows how procuring sleep was an essential part of the gendered labour regimens of household life, as well as a practice that reflected the diverse sites and agents of material innovation in early modern England.^19^

## RECIPE COLLECTIONS

Early modern England was gripped by recipe fever. In this period, an unprecedented number of manuscript and printed recipe collections brought together a broad range of medical, culinary and practical recipes.^20^ Such recipes typically comprised written instructions that recommended the best ways to source, prepare, and combine diverse materials to produce objects, substances, foodstuffs, and medicines. They were usually based on experimental trials, and they were frequently adapted and passed on, often across several generations of a family. Since these collections also reflected a family’s social networks, they were continuously added to and amended by various hands, and thus represented ‘living manuscripts’ rather than static resources.^21^ As Elaine Leong has shown, the production and circulation of recipes lay at the heart of ‘household science’, or home-based investigations of the natural world, in these years.^22^ Personal recommendations from trusted friends and family were especially valued, and recipes which had been tried and tested would frequently be validated with the statement *Probatum est* (‘it has been proven’) as further declaration of their credibility and efficacy.^23^ Details about a collection’s creators and users are often limited, and indeed it seems unlikely that all recipes collected would have been tested.^24^ Nevertheless, evidence from marginal annotations and biochemical fingerprints, as well as the medical inventories of recipe book compilers, suggests that a significant proportion of recipes contained in such collections were recreated in early modern households.^25^

Our recipe sample is drawn primarily from manuscript recipe collections, with the addition of some printed sources. There was considerable correspondence between these two bodies of evidence; compilers of manuscript collections frequently copied out instructions from printed works as well as exchanging manuscript recipes.^26^ The repetition and reworking of specific recipes across contemporary sources reveals significant crossover between print and manuscript.^27^ Householders used their manuscript collections in conjunction with printed works, including recipe books as well as herbals, pharmacopoeias, health regimens and books of simples. Alongside her collection of culinary and medical recipes, for example, gentlewoman Elizabeth Freke took extensive notes from the well-known *Herball* of John Gerard (1545–1612), first published in 1597. Gerard’s *Herball*, and others like it, combined detailed physical descriptions of plants with recommendations about their culinary and medicinal applications.^28^ Printed recipe books entered numerous editions in Latin and vernacular translations, ensuring that recipe culture became a pan-European phenomenon.^29^ Nevertheless, England was a particularly vibrant centre for recipe knowledge, resulting in an abundance of extant manuscript collections compiled by women and men.^30^ Sleep remedies pervade such collections. In a survey of fifty recipe books from the period 1650–1750, for example, eighty percent contained at least one recipe aimed at securing a peaceful night’s rest.^31^ Householders thus anticipated periods of sleep loss through the collection of recipes containing a wide range of ingredients which could be ingested or applied to different areas of the body to encourage sleep.

Women’s agency in compiling, exchanging and testing medical recipes has been foregrounded in existing scholarship. Women played an active role in dispensing advice and aid among their female social networks, while gentlewomen and female servants collaborated in the production of household remedies.^32^ Furthermore, Wendy Wall suggests that England saw the first printed recipe books marketed specifically to women, which covered a range of subjects including herbal cultivation, textile making, medical care and food preservation under the banner of ‘housewifery’.^33^ This publishing strategy points to women’s active participation in recipe design, testing, and application within household settings. Recipe collection was not a distinctly feminine pursuit, and the majority of early modern published recipe collections were created by men.^34^ Nevertheless, through the creation and exchange of recipes, women contributed towards the construction and dissemination of medical and scientific knowledge at a time when they had few other opportunities to do so. Both women and men exchanged recipe knowledge and worked together to compile collections, selecting recipes to include on the basis of an author’s perceived trustworthiness rather than their gender. Robert Boyle’s collaborations with his sister, Katherine Jones, Lady Ranelagh (1615–1691), and sister-in-law, Margaret Boyle, on matters of medical and natural philosophical significance is a prime example of this.^35^ In this article, therefore, we examine sleep recipes collected by both women and men, while paying attention to the ways in which gender shaped their use within the household.

## HERBALS AND SOPORIFIC KNOWLEDGE

Like recipe books, vernacular printed herbals flourished in sixteenth and seventeenth-century England.^36^ The market for botanical books was sufficiently lucrative for publishers to commission and invest heavily in the production of new herbals, which described and sometimes illustrated medicinal plants and related recipes.^37^ These compendia served as practical guides to foraging and cultivation practices, and as functional aids to recipe-making within the household.^38^ Women and men engaged with printed herbals to be guided by medical authority but also to trial, adapt, and personalize the advice that they found according to the circumstances of their own lives.^39^ Indeed, the authors of early modern herbals frequently ‘anticipated a botanically literate audience’ among an engaged readership, which was diverse in composition and attuned to the culture of medical experiment fostered by the material Renaissance.^40^

**Figure 1:**
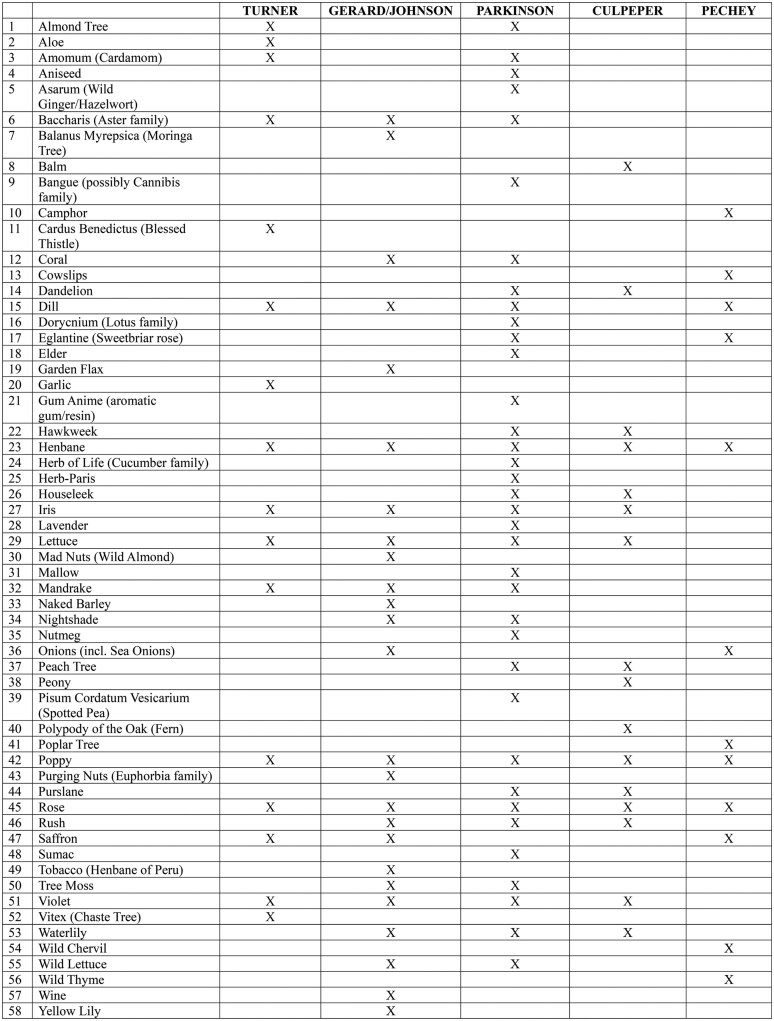
This table shows the fifty-eight plant-based soporifics that appear in the five printed herbals used in this article, and which publication they feature in. Spellings have been modernized and English translations of Latin botanical names have been offered where possible.

Printed herbals ranged from simple pocket-sized octavo volumes explaining the use of plants, including some that focused on plants in use among Indigenous peoples, to large-scale illustrated folios that blended botanical findings from the old and new worlds. Herbals were read, heard, annotated, abbreviated, and gifted as cherished objects in wills by women and men of the middling sorts, by elites, and by aristocrats; as such, their history is inextricably bound up with early modern medical knowledge and practice. Amongst the most well-known and widely-circulating herbals of the period were William Turner’s *The First and Seconde Partes of the Herbal of William Turner* (1568), John Gerard’s (and Thomas Johnson’s) *Herbal or Generall Historie of Plantes* (1633), John Parkinson’s *Theatrum Botanicum: The Theater of Plants* (1640), Nicholas Culpeper’s *The English Physitian Enlarged* (1653), and John Pechey’s *The Compleat Herbal of Physical Plants* (1694).^41^ These works form the basis of our analysis of botanical soporifics, illustrated in Figure 1, which identifies fifty-eight distinct plant species that were credited with soporific powers.

The herbals of Turner (1509/10–1568), Gerard, and Parkinson (1567–1650) were expansive and expensive folios that were widely considered as canonical works, thanks in part to their size and to the prominent roles that their authors occupied within courtly and elite circles. John Gerard, for example, was elected Master of the Barber-Surgeons’ Company in 1596, and his herbal ran to 1,400 pages in length. John Parkinson served at the courts of King James I and King Charles I, and he also made significant contributions to the *Pharmacopoeia Londinensis* (1618), which was published by the Royal College of Physicians in an attempt to standardize English pharmaceutical practices. A copy of Parkinson’s work sold in 1640 for thirty-six shillings, minus the cost of the binding. Such a cost was out of reach for many, but original and adapted versions of these men’s works populated the personal libraries of physicians and apothecaries, and they were also used to support the alchemical experiments of wealthy lay healers such as Margaret Clifford.^42^ By contrast, Nicholas Culpeper’s (1616–1654) best-selling herbal, *The English Physitian*, was a modest quarto volume that could be purchased for just three pence. Culpeper’s work drew heavily on Parkinson’s *Theatrum Botanicum*, but he adapted Parkinson’s entries to ensure that his own text was accessible to a much wider audience.^43^

Botanical soporifics featured prominently in this collection of popular and widely-circulating books. Most of the plants used to procure sleep were believed to work through their cooling and moistening properties, with different elements of each plant varying in temperature, potency, and therapeutic value. The medical efficacy of these plants was also calibrated according to habitat, with most growing in moist and/or shady environments and thought to partake of those conditions. Habitat variation also meant that careful attention was paid to the respective degree of coldness discharged by different plants. Certain varieties of rush, for example, which grew in ‘standing pooles’ and ‘by rivers sides’ had seeds that were considered so cold that they could only be administered with extreme caution, or once tempered by a hot ingredient. Such safeguards were essential to avoid inducing a ‘dead sleepe’ from which the patient might never awake.^44^ Makers of domestic sleep recipes were clearly well acquainted with the material qualities of different soporific plants and their therapeutic principles; especially potent ingredients like nightshade and rushes were used sparingly, in highly tempered combinations, or were accompanied with very precise dosage instructions.

The sleep therapies found in early modern herbals were rooted in botanical observations from classical antiquity and from the early medieval Islamic world, but they also record a more contemporary process of material and medical transformation. The known range of botanic medicaments, including those with soporific power, expanded significantly in this period as knowledge of new plant species emerged through processes of exploration, colonization, and burgeoning global trade networks.^45^ The herbal of Gerard and Johnson, for example, made space for ‘Tabaco, or Henbane of Peru’, which had come to Europe from America’s provinces and from the island of Trinidad. This plant had adapted well to English soils, which suggested to Gerard and Johnson that the home-grown product was ‘better for the constitution of our [English] bodies’ than the ‘Tabaco’ that was grown overseas and imported.^46^ Whatever its source, Gerard and Johnson were struck by the plant’s ‘benumbing qualitie’, which when smoked produced ‘an infirmitie like unto drunkennesse, and many sleepe; as after the taking of Opium’. A strong tone of moral censure often accompanied such observations of Indigenous practice, especially when ‘Tabaco’ was smoked, yet Indigenous medical knowledge of the plant’s soporific effects still guided European practices. Drinking four ounces of the plant’s juice was said, for example, to produce ‘a long and sound sleepe’ that had cured ‘a strong Countreyman of a middle age’ of dropsy.^47^

Four more botanical soporifics originating from America and the Caribbean islands caught the attention of apothecary John Parkinson. Parkinson’s *Theatrum Botanicum* recorded a new variety of Sleepy Nightshade that grew in Virginia, New England, and the West Indies. Parkinson had personally experimented with the seeds and plants of the ‘Virginia Nightshade’ to uncover its medicinal effects.^48^ He also described the virtues of a Virginian violet that was effective in medicinal plasters to ease headaches and procure sleep.^49^ The third plant, which Parkinson termed ‘The Virginia Asarum,’ (or Arabacca) was a species of wild ginger with a heady and spicy scent. The bruised leaves of established varieties of Asarum were known to ease headaches ‘and procureth sleepe’ when applied to the forehead and temples. Parkinson ventured that the Virginian variety ‘is probable to be of the like effects, being so much more aromaticall and sweet’, although he acknowledged that this plant ‘hath beene but little experienced by any that I know’.^50^ The fourth new species was a tree called ‘Virginia Sumacke’. Known varieties of sumac were used to treat ‘want of sleepe,’ whilst also emitting a ‘good sent’ to clothing and household textiles when laid in wardrobes, chests, and presses. As with the Virginia Asarum, Parkinson had not personally experimented with Virginia Sumacke, but he thought it likely that ‘it might worke some of these [similar] effects if any would make the tryall’, or perhaps even supersede existing varieties by avoiding their inconvenient side-effects.^51^ His words appear to acknowledge, and even encourage, a culture of active experimentation amongst his readers – a culture that our recipe sample also illuminates within domestic households. Increasing knowledge of new *materia medica* linked to sleep expanded opportunities for therapeutic intervention through the inclusion and combination of new substances and scents within soporific remedies.

Our recipe sample shows a significant overlap between the soporific plants recommended in the herbals and those selected for home-use. Householders used manuscript recipe collections in conjunction with printed books including herbals, pharmacopoeia, health regimens and books of simples. Mild soporifics recommended in herbal literature, such as lettuce and houseleek, appear repeatedly in domestic recipe collections, probably due to their accessibility and affordability; these ingredients could be easily and cheaply acquired through foraging, cultivation, or purchase.

There is, nevertheless, significant divergence between these two bodies of evidence. Our recipe sample contains several ingredients that were not associated with sleep in the herbal literature, or that merited no mention in these texts; some examples include ivy, mulberry leaves, bean-flower, cumin and walnut leaves. This divergence, we suggest, is evidence of active material experimentation with botanical soporifics in household settings. Recipe-makers drew on the knowledge and advice of herbalists, but they also trialled different ingredients, and new combinations of ingredients, to ensure that their sleep recipes addressed the bodily needs of their own household and the specific illnesses that impaired the sleep of its members. Considerations of thrift must also have shaped recipe-design and making, since householders were encouraged to make the most of what they already had, or what they could gather in close proximity to their homes, before purchasing new goods.^52^

Juxtaposing our sample of household sleep recipes with contemporary herbal advice exposes practices of domestic experiment and reveals the particular health challenges that might demand a sleep therapy. Such insights are often difficult to glean from the manuscript recipe books alone, which give little contextual detail about when and why particular recipes were used. Most of the plant soporifics in our sample were simply noted to procure sleep, but it is unlikely that they were all designed to relieve endemic sleep disorders. These recipes were probably called upon when sleep became troublesome due to a whole host of concerns including common seasonal conditions such as coughs, colds, and lung problems, as well as aches, pains, fevers, and melancholy. The herbal literature shows that a significant proportion of botanical soporifics were also deemed effective in relieving these health problems. Nicholas Culpeper, for example, observed that powdered iris helped to remove catarrh and if ‘put into the Nostrils it procureth sneezing, and therby purgeth the Head of flegm’.^53^ The headaches and earaches that often accompanied congestive illnesses could also find relief though external applications of sweet-smelling plasters and ointments containing roses, lettuce, and wild thyme, which had well-established soporific effects.^54^ Fevers or ‘agues’ often went hand-in-hand with seasonal infections, or resulted from place-based conditions such as malaria (formerly ‘ague’ or ‘marsh fever’). Whatever the cause, patients sought relief in established soporifics like waterlily flowers, whose subtly sweet scent Culpeper recommended ‘to procure rest, and to settle the Brains of Frantick persons’, by cooling the ‘hot distemperature of the Head’.^55^ Soporific plants were also judged beneficial in the herbals for relieving inflammation of the eyes, joints, and stomach pain, and for treating melancholy. All of these commonplace medical conditions had the capacity to prevent or disrupt restful sleep in different ways; and restful sleep, in turn, helped to alleviate them. By identifying the wide range of illnesses that affected sleep, or were relieved by peaceful rest, herbals also help us to better understand the vital importance of sleep-inducing recipes in household medical care. It is to those practices of household care that we now turn.

## RECIPES, SLEEP AND THE SENSES

Evidence from early modern recipe collections reveals that the manipulation of the senses was of the utmost importance for sleep care. The creation of sleep remedies depended on the sensory knowledge of materials, including an awareness of how combinations of ingredients affected the bodies of those who touched, tasted and smelled them. The importance of the senses for inducing sleep drew on longstanding Aristotelian theories of sensation, in which the ‘species’ of animal, mineral and organic materials physically acted upon a person’s sense organs, moving the body’s sensible spirits and triggering a bodily response.^56^ The calming and incapacitation of the senses was held to be necessary for sleep. For Aristotle this was achieved through the digestive process, which cooled the upper parts of the body and concentrated heat around the heart.^57^ Early modern writers identified a range of other ways to calm the senses for rest. English philosopher Francis Bacon (1561–1626), whose conception of scientific observation profoundly influenced the development of experimental scientific methods, suggested that sounds such as ‘the blowing of the Wind, the trickling of Water, humming of Bees, soft singing, reading’ would encourage sleep since they ‘[still] the natural and discursive motion of the Spirits’.^58^ In his commonplace book of c. 1630, Lancashire recusant and antiquary Christopher Towneley (1604–1674) similarly noted that sleep’s ‘formall cause’ was ‘the rest of the outward senses, viz. sensing & seeing’.^59^ He suggested that this rest could be attained by applying or ingesting soporifics that drew heat to the body’s centre and moistened the brain, while calming the motion of the animal spirits, which were produced in the brain and moved between mind and body.^60^ To achieve such results, Towneley’s manuscript contained a number of soporific recipes, including a cooling mixture of rose oil and ‘womans milke’ to be applied to the temples. This recipe followed Towneley’s physiological understanding of sleep, since the cooling of the extremities – especially the head and feet – was thought to encourage the concentration of heat at the centre of the body, thereby inducing rest.^61^ In combination with domestic experimentation, medical theories of sleep and sensation thus informed the development and application of sleep therapeutics.

The significance of the extremities for sleep’s process is also reflected in the number of soporific recipes which focused on the feet. Thomas Sheppey’s (b. 1641/2) recipe book included instructions to boil leaves of the cooling white willow and ‘wash thy feet therewith’.^62^ Contemporary collections also included recipes for anointing the soles of the feet with water distilled from the mallow plant, or even roasted onions.^63^ While not necessarily cooling, these botanicals were recognized for their soporific qualities, which were conveyed through targeted tactile application to the feet.^64^ A more unusual recipe from the turn of the eighteenth century recommended preparing two socks of sheep’s leather spread with a kind of plaster (‘*implastrum septialicum*’) to be worn ‘till they fall off’, at which point the brain would be settled and restored in preparation for rest.^65^ In this recipe calming sensory manipulation of the feet has a direct impact on the brain, highlighting the interconnected significance of the body’s extremities for the physiological onset of sleep.

While recipes focused on the feet are prominent in manuscript collections, our recipe sample nevertheless reveals an overwhelming preference for cooling tactile remedies which centred on the patient’s head. A recipe book compiled c. 1625, for example, recommended preparing a ‘plaister’ of leavened wheat bread topped with rosewater, powdered rose leaves and rose oil to lay over the head and temples. This recipe thus combined the sensory manipulation of cooling touch and the sweet, soporific scent of roses. A marginal annotation in a later hand suggested adding ‘womans milke’, hinting that the recipe had been tested and found to benefit from this addition.^66^ Another soporific remedy in the same collection instructed the maker to ‘Take the joyce of house leeke woomans milke rosewater oyle of Roses and putt therein a peece of a rose cake and laye yt between tow cloathes and binde yt to the foreparte of the heade’.^67^ Both of these recipes acted on the principle of cooling the head to induce rest; when applied to the forehead and temples, cooling botanicals like rose and houseleek were understood to penetrate the skin and calm the brain, a prerequisite for sleep’s onset following Aristotelian conceptions of digestion.^68^ John Parkinson noted that the cooling houseleek could also treat hot agues, headaches and the ‘distempered heate of the braine in frensyes or through want of sleepe’, offering a prime example of how the soporific properties of botanical ingredients related to the treatment of other ailments which were often similarly connected to the temperature of the head and brain.^69^ Besides calming the mind and spirits, these remedies may also have worked to procure sleep by combatting sources of pain or discomfort which prevented peaceful rest. Thomas Carter’s recipe collection from 1621, for instance, included several recipes ‘to lay to the Head when one is in a burning Ague and cannot sleep’.^70^ The connection between securing healthy sleep and the treatment of common illnesses probably elevated the value of soporific recipes for householders, particularly those which focused on the head.

The head and brain were critical sites of soporific treatment throughout the early modern period, and they grew in significance in the seventeenth and eighteenth centuries as new neurological models of sleeping and waking were developed in connection with anatomical investigations of the brain.^71^ As recognition of the brain’s importance for sleep increased, so too did the number of soporific recipes which functioned through the sense of smell. In addition to being critical to peaceful sleep and dreams, prior to 1700 the brain was widely accepted to be the organ of olfaction.^72^ Rather than registering and evaluating a scent, the nose and nostrils merely carried smells to the brain.^73^ Smells themselves originated from the spirits present in all things, both animate and inanimate.^74^ During olfaction, particles of these odorous spirits were transferred physically to the brain where they had the capacity to comfort or disturb the bodily spirits which governed sleep; scents thus had a profound physiological impact on people’s ability to sleep soundly. Acceptance of the embodied effects of scent gradually declined across the eighteenth century, meaning that – in combination with the heightened importance of the brain for sleep – the period between the mid seventeenth and early eighteenth centuries was a particularly significant moment for the use of smells in sleep remedies.

Of all the senses, therefore, smell was the most frequently appealed to in soporific recipes in this half century. Countess of Kent Elizabeth Grey’s (1582–1651) extremely popular recipe book, for example, first published in 1653, contained instructions on how to make a ‘bag to smell’ with powdered rose leaves, mint and cloves which you should take ‘to bed with you and it will cause you to sleep’.^75^ Roses possessed a cooling scent, which would calm the mind, senses, and spirits, while mint and cloves, which were both warm and dry, could assist with headaches, aid digestion and comfort the heart.^76^ This recipe was carefully copied out in Henry Pagett’s manuscript cookbook later in the seventeenth century, including the instruction to store the rose leaves ‘close in a glasse which will keepe them sweete’.^77^ The sweetness of roses, in addition to their coolness, was particularly important for promoting rest. Sweet smells, most often understood as scents which were pleasant or agreeable in this period (as opposed to sugary or saccharine), were thought to soothe and quiet the brain. In so doing, they facilitated rest and helped to rejuvenate the vital and animal spirits. Meanwhile unpleasant smells were known to disrupt rest, prevent sleep and dull the spirits.^78^ Since the refreshment of the animal spirits within the brain was a key element in neurological models of sleep, scents which assisted with this process were particularly useful for procuring healthy rest.^79^ John Floyer (1649–1734) noted that the scent of cowslips induced sleep by ‘refreshing the Spirits more than stupefying; or rather by a sweet headiness, overcoming the Spirits’.^80^ Sweetness, which ‘overcame’ the animal spirits, was a recurring feature of soporifics, included in recipes centred around both senses of smell and taste.^81^

The properties of coolness and sweetness were characteristic of the range of botanical ingredients used in one of the most popular and widely reproduced soporific recipes in our recipe sample. This recipe appears in at least five manuscript recipe collections dating from the early seventeenth to early eighteenth centuries, and was reprinted in editions of Culpeper’s *School of Physick* from 1659 onwards.^82^ The version in Thomas Sheppey’s manuscript recipe book (1675) instructs the reader to mix ‘Juice of Henbane, Lettice, plantane, poppy, mandrake leaves, Ivy and Mulberry leaves’, which should then be soaked up by a sponge and dried in the sun. Sheppey wrote, ‘when you would have any body sleep, lay the spunge at his nose’, indicating universal effectiveness.^83^ To wake the sleeper, one should put a sponge soaked in vinegar to their nostrils and the sharp scent would cause them to awake. In his collection, Charles Leighton wrote of this recipe that ‘this is an excellent thing & true’, suggesting that he may have tried it himself or been reassured of its usefulness by an acquaintance.^84^ The recipe’s ingredients vary slightly across extant versions – several manuscripts included opium among the other botanicals listed by Sheppey, including a c. 1600 copy of John de Feckenham’s (c. 1515–1584) book which contains the earliest reference to such instructions we have found. Rather than being suitable for ‘any body’, this recipe was said to cause sleep specifically in those who were ‘frantick’, probably suffering from frenzy, and thus in a heightened state of agitation and emotional excitement.^85^ That later authors expanded the target audience of this recipe beyond those suffering from the intense inflammation of the brain in frenzy may reflect the heightened importance assigned to the cooling and calming of the brain for all those seeking rest from the mid-seventeenth century onwards.

While sweet and cooling scents were understood to induce sleep in general due to their effects on the brain and spirits, contemporaries nevertheless recognized that embodied differences might determine the impact of a particular smell on an individual’s mind and body. Scottish clergyman and writer Alexander Ross (1590–1654), for example, noted that for some, roses ‘may bee offensive’, particularly to those whose brains were ‘extraordinary cold, some extraordinary dry, and whose olfactive passages are wider than usually’, precisely because of their cooling nature. Since roses were ‘refrigerative’, Ross stated, they ‘may comfort the hot, but not the cold brain. And if the brain be dry, & the passages wide, the smel doth too suddenly affect it’, causing the brain to ache.^86^ Different smells had different degrees of coolness, and thus some soporific scents were preferable to others depending on the heat of a person’s brain. Since not all recipes worked for every individual, a variety of cures using a range of ingredients and methods of application needed to be developed and recorded, further encouraging domestic medical experimentation in relation to sleep and scent.

The diversity of methods of application was a marked feature of new scent-based soporific therapies in the later seventeenth century. Scent-related treatments were administered in several ways. Some recipes involved application directly to the nostrils. A manuscript recipe book from this period, for example, instructs readers to mix ‘womens breast milk’ with aquavitae and ‘moysten therewith the temples of the patient and his nostrills’, using ‘some feather or some fine thin ragg’.^87^ Another volume from the same period suggests mixing finely beaten nutmeg with rose water and anointing ‘the temples & a little within the Nostrills’ to ‘procure sleep and comforte the spiritts’.^88^ Scents could also be held near the nose to secure similar effects, as in the example from the Orrery family recipe book. An anonymous recipe from 1675 that instructed readers how to make ‘a round ball’ of scent to ‘hold…in there hands & smell unto it’ to ‘make them sleepe,’ was perhaps intended for those who wanted greater control over the length of time such scented ingredients were in contact with the nose.^89^ The recipe required wild poppy, mandrake juice, ‘lees of wine’ and civet. Both poppies and mandrakes were recognized soporifics, while lees of wine were the dregs of wine casks and would have bound the ingredients together.^90^ Civet, the glandular secretion of civet cats, was increasingly used for perfuming in early modern England as a result of trade connections in Asia and Africa. It provided an earthy yet sweet animalic scent and, as a ‘fixative’, increased the longevity of other odours.^91^ The range of different scents used in these recipes, in addition to the various methods of application, indicates contemporary experimentation with soporific smells based on the availability of particular materials as well as individual bodily differences.

Recipes for scented soporifics to be held near or applied directly to the nose acted on the understanding that smells had a direct impact on the brain, comforting and calming the mind and spirits.^92^ As physicians increasingly emphasized the positive effects of scents on the brain from the second half of the sixteenth century, Sandra Cavallo suggests that a new range of objects was developed which, rather than purifying the ambient air, were intended to be sniffed by individuals for personal benefit.^93^ The use of such items echoed the increasing production of perfumed accessories such as gloves, buttons and beads which were worn on the body to combat external, pathogenic odours.^94^ The development of all such objects resulted from the boom in material production and consumption with the material Renaissance. This growing emphasis on personal treatment via smell, involving material innovation and experimentation, was reflected in soporific recipes; seventeenth-century recipe writers increasingly advised that scents should be applied to the nose and nostrils (rather than head or feet) to encourage rest. Lay practitioners thus appear to have been aware of the growing importance of the brain in understandings of sleep’s physiology, a development which guided their therapeutic experiments in sleep care.

## SCENT AND SLEEPING ENVIRONMENTS

Besides direct application to the nose scents were applied to sleeping environments, including beds, bedding and chambers, to ensure healthy sleep. This broader use of scent for healthy sleep drew on the perceived impact of smells on the brain, as well as their association with air quality. It was widely agreed in contemporary health guidance that healthy sleeping environments should be well ventilated with fresh, ‘sweet’ and clean air which would protect the body’s internal functions, remove putrid vapours, and encourage peaceful sleep.^95^ If a sleeping place lacked sufficient air circulation, unpleasant smells could disturb the mind and disrupt one’s rest. Norfolk clergyman James Woodforde (1740–1803), for instance, recorded that on winter nights, when he had to keep his chamber windows closed, the ‘intolerable smoke and stink’ from his night candles prevented him from sleeping soundly.^96^ The need to control air quality, and therefore the smells which were present within a sleeping environment, encouraged the development of a further range of soporific recipes intended for scenting sleeping places and bedding textiles.

While practices of domestic scenting were informed by medical and botanical literature, which was typically written by men, the majority of recipes for perfuming beds, bedding and chambers appear in manuscript recipe books belonging to middle and upper-class women. The recipe book of Mary Doggett, wife of actor Thomas Doggett (c. 1670–1721), for example, contains at least four recipes for perfuming linen, while Lady Anne Fanshawe’s (1625–1680) collection includes three such recipes.^97^ Further recipes were recorded in volumes by Lady Ann Charlotte Frescheville (ca. 1640–1717), Lady Frances Catchmay (d. 1629), Lady Catherine Fitzgerald, and a Lady Barrett.^98^ Creating and experimenting with scents were commonplace domestic duties among gentlewomen who, with the assistance of their female servants, thus transmuted prescriptive healthcare advice into practical domestic interventions to safeguard the sleep fortunes of their households.

Chambers would be kept ‘sweet’ and fresh through the use of scented botanicals, strewn on the ground or used to perfume beds and linens. Recipes involving such scenting practices in contemporary collections typically focused on the humoral balance of ambient air.^99^ Since sleep required a cooling of the body, smells tended to be selected based on their cooling qualities, in a similar manner to scents which were applied directly to the head and nose. Thomas Dale’s translation of Jodocus Lommius’s (1527?–1572) medical treatise on fevers noted that ‘cooling the air of the chamber’ would help a patient sleep, and could be achieved by ‘wetting the floor with Oxycrate [a mixture of water and vinegar] or Rose-water’ and strewing the room and bed with ‘branches of willow or vine’.^100^ Such instructions regarding the use of cooling botanicals in sleeping chambers were reflected in manuscript recipe books such as that of Lady Frescheville’s from 1669, which contained a recipe for ‘A perfume for a Roome’ involving benjamin (benzoin), civet and spirit of roses.^101^ These ingredients should be mixed together ‘according to your own smell’, thus allowing for flexibility depending on individual bodies and tastes. Once the perfume was ‘as you like it’, the reader should ‘take a little upon a bodkin [and] touch the hanging or the curtaine, or the bed with it, and it will perfume the whole Roome’. Benjamin, a gum or resin sourced from trees in the Styrex genus, had cooling properties and was known to resist malignant humours, bringing particular relief to the brain and chest.^102^ Like civet, benjamin is a fixative – as well as providing its own sweet vanilla-like smell, therefore, it would enhance the other scents with which it was combined.^103^ Though not explicitly described as a soporific recipe, the use of this perfume in a sleeping chamber was almost certainly due to its sleep-inducing qualities; the dominant scent was spirit of roses, and roses were widely understood to encourage rest thanks to their coolness and their ‘sweet and pleasant smell’.^104^

Sweetness was a characteristic feature of soporific ambient smells, as it was for those applied to the face. Contemporary commentators noted that sleeping chambers needed to be ‘sweet and wholesome’ to promote rest.^105^ A scent could nevertheless be ‘too sweet’, in which case it had the potential to disturb the mind and senses. John Gerard wrote of pipe privet flowers, or the common lilac, that they possessed a ‘pleasant sweet smell’ but in his view were ‘too sweet, troubling and molesting the head in a very strange manner’. He noted that he had once placed such flowers in his chamber overnight, where their smell became so overpowering that, they ‘awakened me out of my sleep, so that I could not take any rest till I had cast them out’.^106^ Gerard’s anecdote illustrates a tension that existed between the use of soporific scents in sleeping environments and the view that strong smells could impair sleep quality by agitating the brain.^107^ As the brain gained greater significance in physiological understandings of sleep, the management of ambient smells became more important for rest. Since scents could both comfort and unsettle the mind, it was essential to achieve the right balance of fragrances within a sleeping chamber, necessitating careful experimentation. The dangers of overpowering scents for the brain meant that some considered the best smell for health to be ‘no smell at all’.^108^ Recipes for perfuming sleeping chambers nevertheless abound in contemporary collections, though some compilers deliberately sought to produce subtle scents. For example, a ‘Mrs Carr’ noted down instructions for a ‘delicate perfume for a chamber’ in 1682, which consisted of rose leaves, powder of damask roses, cloves, nutmeg, cinnamon, orange peel, walnut leaves, lemon, thyme and sweet briar (eglantine).^109^ The desire for delicacy was probably connected to the need to avoid overpowering the brain with strong fragrance, and was possibly informed by contemporary discussions concerning the relationship between smell and the brain.

Besides the chamber, the bed itself was a central site for the use of soporific scents. Sheppey’s recipe book, for instance, included a recipe to procure sleep using aniseed which stated simply, ‘lay annis to their pillows, so that they may smell it’.^110^ Similarly, John Pechey’s herbal noted that broken branches of sweet briar, which possessed a ‘curious smell’, when ‘laid on the pillow, disposes to sleep’.^111^ Pillowcases and cushions could be filled with scented botanicals, like hops or roses, to lull the senses to sleep.^112^ In 1573 John Partridge (fl. 1566–1582) instructed readers to ‘gather red Roses in faire wether’ which should be dried out and then placed in a pillowcase and laid ‘vpon your bed between the Couerlet and the Blancket, all night’.^113^ Recipes for perfuming linen were particularly popular in early modern recipe collections, taking the form of scented powders, waters and ‘sweet bags’. One anonymous manuscript included instructions for mixing damask rose water, lavender, benjamin, ‘laudam’, orris roots (from the Florentine iris), musk and civet, which should be sprinkled onto linen as it was folded for storage.^114^ This is one of several examples from our recipe sample in which lavender is used as a soporific scent.^115^ The sleep-inducing properties of lavender were recognized in this period, but it was far from the dominant sleep-related scent that it is today. Rather, lavender was viewed as one among many scented botanicals which could encourage rest. This diversity of soporific smells contributed to the great variety of sleep care recipes which operated through scent. While the compiler of this particular collection is unknown, the laundering and folding of linen was a task predominantly undertaken by women, who would thus have played a key role in the scenting of bedding materials for healthy sleep.

Sweet bags were also typically made by women, and consisted of highly decorative, pouches of scent which could be placed alongside household textiles in storage, tied to the bedstead or even worn on the person.^116^ The same anonymous collection discussed above contained a recipe for sweet bags involving the ‘mosse of sweet apple trees’, which should be left to soak up damask rose water before being dried out and mixed with benjamin, storax and calamite, dried rose leaves, musk, civet, ambergris, oil of lemons, and orange flower water. It instructed: ‘work all these with yr hands into ye mosse’ which should then be placed in rosewater and dried out again before being deposited in ‘silk baggs’. The recipe promised that this ‘will continue a most excelent smell 20 years’.^117^ In this case it seems likely that a potential maker would have bought exotic ingredients like benjamin, musk, civet and ambergris from apothecaries or druggists, which they would then have mixed with moss they had collected themselves. Moss was known to be highly absorbent and would thus have acted as a sponge for these scents. Commentators also noted that tree moss itself possessed soporific qualities.^118^

Early modern women thus engaged in hands-on experimentation with a wide range of local and global ingredients to produce perfumes aimed at ‘sweetening’ beds and chambers and thereby encouraging rest. In addition to a deep well of botanical knowledge, contemporary inventories provide evidence that women possessed the technical equipment, and therefore the practical skills, to prepare domestic perfumes. Among Arthur Coke of Branfield’s (1588–1629) listed possessions in 1629 were ‘a perfuming pan and bason’ which were stored in his wife’s closet.^119^ Women were at the forefront of domestic experiments with soporific perfuming recipes, which were embedded within wider cultures of domestic medical experimentation.

The importance of scenting domestic spaces was connected to broader conceptions of the entanglements between sleep, health and environments. The intrusion of outside airs into sleep chambers was a source of concern. It was believed, for example, that harmful vapours, kept at bay by the warmth of the sun, would descend upon sleeping bodies, causing sickness and disease. Writing of the harmful effects of smoke pollution in early modern London, author and diarist John Evelyn (1620–1706) commented that smoke, which ascended into the atmosphere during the day, would descend again with the coolness of nighttime, when it would be absorbed by ‘our houses, the waters, and … our Bodies’.^120^ The same was understood of infectious air, or ‘miasma’, which was typically understood to be foul smelling.^121^ Such views of the effects of unhealthy or polluted air on sleeping bodies, which appear in texts concerning the management of local environments for health, were also reflected in contemporary recipe collections for application in the home. Lady Francis Catchmay’s book of medical recipes from the late seventeenth century included a perfume of St John’s wort, olibanum, mastic and juniper which would ‘preserue mankind from the infection of the plague’, and was to be laid ‘on a hot tyle either in yor Chamber or in the parlor beneathe where you goe to bed at nighte’.^122^ The heat of the tile would cause the scent to gently disperse and rise into the sleeper’s chamber above. The sleeping body was at particular risk from infectious vapours, not only due to their predominance in the night air, but due to the heightened bodily vulnerability of sleep.^123^ Methods of cleansing the air at night, chiefly through scent, were thus especially important for both sleep and health more generally.

Also responding to the ‘infectious ayr of London’, in 1680 an anonymous author recommended an unusual means of protecting sleeping bodies using scented botanicals. They wrote that it had become custom ‘with those at Oxford to Aire their Rooms with the sweet vapour’ of juniper, the smoke and scent of which was known to drive away ‘infection and corruption of the air’.^124^ For London, however, the author recommended a longer-lasting method of freshening one’s chamber. This involved plastering and then painting the ‘walls of our Lodging Rooms’ with the ‘Chimical Oiles or Essences, of Mother of Thyme, Sweet Majoran, Musck [and] Roses’. Not only would this result in a lasting scent, but it would ‘mightily correct any curde & cold vapours which may be suspected to throw themselves from the wall in damp or very cold season’.^125^ Such scents would thus help to protect sleepers from the dangerous vapours of the night air. This novel proposal, within a broader (unpublished) pamphlet outlining methods to counter the city’s pollution, indicates how material experimentations with smell in relation to sleep were intertwined with larger efforts aimed at ‘improving’ environments deemed to be unhealthy.

In the same text in which he warned against the nightly precipitating smoke of London, Evelyn outlined a plan for improving the city which involved planting acres of land with ‘fragrant and odoriferous flowers’, whose ‘redolent and agreeable emissions’ of scent would ‘perfectly improve and meliorate the Aer’.^126^ Among these were numerous botanical ingredients used throughout soporific recipes involving scent, including roses, violets, juniper, sweet briar, lemon, mint and thyme. William Cavert has argued that Evelyn’s text, rather than an early environmental manifesto, was the culmination of existing traditions which sought to improve and reform the city’s air.^127^ Our investigation of early modern recipes for scenting the home indicates that such methods of environmental improvement also had roots in domestic health care practices, performed chiefly by women, in which sleep was vitally important.

## CONCLUSION

Procuring sleep was a central concern driving domestic medical experimentation in early modern England. Women and men attempted to secure healthy sleep using a broad range of recipes and ingredients, in combination with a growing array of printed botanical and medical literature including herbals, pharmacopoeia, and health regimens. The high stakes of sleeping well ensured it became a critical therapeutic issue in household medicine. Obtaining the right amount of sleep was understood to be essential for overall bodily health; sleep was also a crucial component in medical treatments aimed at numerous commonplace illnesses, including fevers, headaches and congestion, which typically centred on the head and brain. Given sleep’s vital importance for everyday practices of health management, it is little wonder that the increasing number of women and men who experimented with creating medicinal treatments at home focused their efforts on securing peaceful sleep.

Such experimentation was encouraged by the wide and increasing range of available interventions to support sleep, which continued to expand across the sixteenth and seventeenth centuries with the impact of the material Renaissance. Analysis of our recipe sample, in combination with contemporary herbal literature, has uncovered far more diversity in botanical soporifics than has so far been realized. The number of recognized soporifics increased in this period in connection with the expansion of herbal knowledge through global trade, colonialism and exploration. In addition to embodied differences and sleep-preventing ailments, the varied ingredients chosen for sleep remedies were constrained by budget and locality: while some, such as civet and benjamin, were expensive imports purchased from apothecaries or druggists, others would be cultivated or foraged locally. The practices of domestic experimentation reflected in manuscript recipe books also encouraged the incorporation of botanicals which were not recognized by contemporary herbalists. Such practices of collecting and testing diverse recipes and ingredients were motivated by recognition of the embodied differences between individuals, since a person’s humoral balance determined the effectiveness of a particular remedy for their body and mind. As sleep loss was a serious health concern, with the potential to affect all members of a household, individual bodily differences required the pre-emptive compilation of multiple soporific recipes.

Given the importance of the senses within physiological explanations of sleep’s onset, householders were able to experiment with different modes of application involving sleep-inducing ingredients. In particular, this article has highlighted the critical importance of scent therapeutics, in connection with the increasing emphasis placed on the brain and nerves for sleep’s operation from the mid seventeenth century onwards. This shift in physiological knowledge alongside the growing number of available soporific ingredients encouraged the development of recipes for application to both bodies and sleep environments. The domestic context of these interventions offers clear evidence of women’s engagement in the changing therapeutics of sleep; the perfuming of textiles and chambers aligned closely with women’s domestic labour and expertise. Early modern women thus translated prescriptive advice concerning the interconnections of scent, air quality, health and sleep into everyday healthcare practices.

The plethora of sleep-related recipes recorded in manuscript and printed recipe books from the sixteenth century onwards marks a notable shift in sleep habits, in accordance with the greater engagement with matter and processes of making that characterized the material Renaissance. The well-known argument that sleep patterns changed radically between the early modern and modern periods – shifting from biphasic to consolidated sleep – is not directly supported by our findings; none of the recipes in our sample reference segmented sleep (by indicating that a remedy should be applied in between sleep intervals, for example) and instead appear to aim for wholly undisturbed rest.^128^ Our research makes clear, however, that a transformation in sleep habits involving new practices of bodily labour and material innovation took place prior to modern industrialization. In the vibrant culture of practical and intellectual enquiry which took shape from the early sixteenth century, early modern people were encouraged to invest unprecedented amounts of time, labour and money into the production of sleep remedies. Extensive processes of trial and error centred around this single quotidian issue – of sleeping well – which was nevertheless understood to determine the health of all early modern people. This article firmly positions soporific recipe-making within the broader culture of the material Renaissance, and in doing so it expands our view of ‘who had a Renaissance and how’.^129^ The Renaissance initiated new patterns of consumption and technological advancements which depended on contemporaries’ imaginative engagement with matter. Yet such material engagement also transformed daily habits and experiences as fundamental to early modern life as the very act of going to sleep.

## Footnotes

1 ‘Boyle Family Recipe Book’, MS.1340, Wellcome Library (hereafter WL), c.1675–c.1710, fol. 122v.

2 Authorship of this manuscript has been contested, but our attribution follows Michelle DiMeo, ‘Lady Ranelagh’s Book of Kitchen-Physick?: Reattributing Authorship for Wellcome Library MS 1340’, *Huntington Library Quarterly* 77: 3, 2014, pp. 331–46.

3 Robert Boyle, ‘An account of the two sorts of the Helmontian Laudanum, communicated to the publisher by the Honourable Robert Boyle, together with the way of the Noble Baron F.M. van Helmont (son to the famous Johannes Baptista,) of preparing his Laudanum’, *Philosophical Transactions of the Royal Society* 9: 107, 1674, pp. 147–9.

4 DiMeo, ‘Lady Ranelagh’s Book of Kitchen-Physick?,’ p. 340.

5 ‘Boyle Family Recipe Book’, MS.1340, WL, fols. 17r, 44r, 121v, 122r, 132v, 133v; *Spirits Unseen: the Representation of Subtle Bodies in Early Modern European Culture*, ed. Christine Göttler and Wolfgang Neuber, Leiden 2007; Penelope Gouk, ‘Music and Spirit in Early Modern Thought’, in *Emotions and Health, 1200–1700*, ed. Elena Carrera, Leiden 2013, pp. 221–39; Hannah Newton, *From Misery to Mirth: Recovery from Illness in Early Modern England*, Oxford 2018, pp. 76–7; Bill Maclehose, ‘Fear, Fantasy and Sleep in Medieval Medicine’, in *Emotions and Health*, ed. Carrera, pp. 67–94, 83.

6 Thomas Cogan, *The haven of health chiefly gathered for the comfort of students*, London 1636, p. 269.

7 Sandra Cavallo and Tessa Storey, *Healthy Living in Late Renaissance Italy*, Oxford 2013, pp. 3-7; Sasha Handley, *Sleep in Early Modern England*, New Haven and London 2016, pp. 20–22.

8 Mark S.R. Jenner and Patrick Wallis, ‘The Medical Marketplace,’ in *Medicine and the Market in England and its Colonies c.1450–c.1850*, ed. Mark S.R Jenner and Patrick Wallis, Basingstoke 2007, pp. 1–23; Mary E. Fissell, ‘The Marketplace of Print,’ in Jenner and Wallis, *Medicine and the Market*, pp. 108–32. See also Mary Fissell, ‘Popular Medical Writing,’ in *The Oxford History of Popular Print Culture*, vol. 1: *Cheap Print in Britain and Ireland to 1660*, ed. Joad Raymond, Oxford 2011, pp. 417–30; Paul Slack, ‘Mirrors of Health and Treasures of Poor Men: The Uses of the Vernacular Medical Literature of Tudor England,’ in *Health, Medicine and Mortality in the Sixteenth Century*, ed. Charles Webster, Cambridge 1979, pp. 237–73.

9 Important exceptions include Francesca Elizabeth Richards, ‘ “The Advice of a Gent Who Died from Neglecting it”: The Gentlemanly Pursuit of Knowledge Regarding Domestic Medicine in Kent c.1630–1800’, *Social History of Medicine*, 2024; Abigail Greenall, ‘Nurturing Happiness: Affective Health and Wellbeing in the North West of England, c. 1550–1700,’ unpublished PhD Thesis, University of Manchester, 2023.

10 Elizabeth K. Hunter, ‘‘‘To Cause Sleepe Safe and Shure”: Dangerous Substances, Sleep Medicine and Poison Theories in Early Modern England’, *Social History of Medicine* 35: 2, 2022, pp. 473–93.

11 Anne Stobart, *Household Medicine in Seventeenth-Century England*, London 2016; Jennifer Evans and Sara Read, *Maladies and Medicine: Exploring Health & Healing 1540*–*1740*, Barnsley: Pen & Sword 2017; Alun Withey, *Physick and the Family: Health, Medicine and Care in Wales, 1600*–*1750*, Manchester 2011; *Reading and Writing Recipe Books, 1550*–*1800*, ed. Michelle DiMeo and Sara Pennell, Manchester 2018. On the global circulation of goods see Beverly Lemire, *Global Trade and the Transformation of Consumer Cultures: The Material World Remade, c.1500*–*1820*, Cambridge 2018.

12 Ulinka Rublack, ‘Matter in the Material Renaissance’, *Past & Present* 219: 1, 2013, pp. 41–85; Pamela H. Smith, *From Lived Experience to the Written Word: Reconstructing Practical Knowledge in the Early Modern World*, Chicago 2022; Pamela H. Smith and Benjamin Schmidt, ‘Knowledge and Its Making in Early Modern Europe,’ in *Making Knowledge in Early Modern Europe: Practices, Objects, and Texts, 1400*–*1800*, ed. Pamela H. Smith and Benjamin Schmidt, Chicago 2007, pp. 1–16.

13 Ulinka Rublack, ‘Renaissance Dress, Cultures of Making, and the Period Eye’, *West 86^th^: A Journal of Decorative Arts, Design History & Material Culture* 23: 1, 2016, pp. 6–34; Pamela H. Smith, *The Body of the Artisan: Art and Experience in the Scientific Revolution*, Chicago 2004; Pamela H. Smith and Tonny Beentjes, ‘Nature and Art, Making and Knowing: Reconstructing Sixteenth-Century Life-Casting Techniques’, *Renaissance Quarterly* 63: 1, 2010, pp. 128–79; Stefan Hanß, ‘Gendering the Material Renaissance: Women, Industriousness and the Female Body at the Court of Württemberg’, *German History* 41: 3, 2023, pp. 367–99; Leonie Hannan, *A Culture of Curiosity: Science in the Eighteenth-Century Home*, Manchester 2023.

14 On the importance of recipes see Elaine Leong, *Recipes and Everyday Knowledge: Medicine, Science, and the Household in Early Modern England*, Chicago 2018; Elaine Leong, ‘Collecting Knowledge for the Family: Recipes, Gender and Practical Knowledge in the Early Modern English Household’, *Centaurus* 55: 2, 2013, pp. 81–103; Stefan Hanß and Umberto Veronesi, ‘“The Lute of Wisdom”: Alchemy, the Body, and Medicine in the Material Renaissance’, *Nuncius* 38: 1, 2023, pp. 1–31; Stefan Hanß, ‘Face-Work: Making Hair Matter in Sixteenth-Century Central Europe’, *Gender & History* 33: 2, 2021, pp. 314–45; Alisha Rankin, *Panacea’s Daughters: Noble Women as Healers in Early Modern Germany*, Chicago 2004; Amanda Herbert, Heather Wolfe, and Elizabeth DeBold, ‘First Chefs: Fame and Foodways from Britain to the Americas’, Folger Shakespeare Library Exhibition (2019): [https://folgerpedia.folger.edu/First_Chefs:_Fame_and_Foodways_from_Britain_to_the_Americas; accessed 3 May 2023]. On the use of natural materials see Ulinka Rublack, ‘Befeathering the European: The Matter of Feathers in the Material Renaissance’, *American Historical Review* 126: 1, 2021, pp. 19–53; Stefan Hanß, ‘Making Featherwork in Early Modern Europe’ in *Materialized Identities: Objects, Affects and Effects in Early Modern Culture, 1450*–*1750*, ed. Susanna Burghartz, Lucas Burkart, Christine Göttler, Ulinka Rublack, Amsterdam 2021, pp. 137–185; *Conchophilia: Shells, Art, and Curiosity in Early Modern Europe*, ed. Marisa Anne Bass, Anne Goldgar, Hanneke Grootenboer, Claudia Swan, Princeton 2021; *Ingenuity in the Making: Matter and Technique in Early Modern Europe*, ed. Richard J. Oosterhoff, José Ramón Marcaida, and Alexander Marr, Pittsburgh 2021. On the historiography of ‘experiment’ see Simon Werrett, *Thrifty Science: Making the Most of Materials in the History of Experiment*, Chicago 2019, pp. 12–13.

15 Elaine Leong and Alisha Rankin, ‘Testing Drugs and Trying Cures: Experiment and Medicine in Medieval and Early Modern Europe’, *Bulletin of the History of Medicine* 91: 2, 2017, pp. 157–82; Rublack, ‘Renaissance Dress’, p. 11. See also Sandra Cavallo and Tessa Storey, *Healthy Living in Late Renaissance Italy*, Oxford 2013; *Conserving Health in Early Modern Culture: Bodies and Environments in Italy and England*, ed. Sandra Cavallo and Tessa Storey, Manchester 2017.

16 Erik A. Heinrichs, ‘The Live Chicken Treatment for Buboes: Trying a Plague Cure in Medieval and Early Modern Europe’, *Bulletin of the History of Medicine* 91: 2, 2017, pp. 210–32; Alisha Rankin, *The Poison Trials: Wonder Drugs, Experiment, and the Battle for Authority in Renaissance Science*, Chicago 2021.

17
*Patients and Practitioners: Lay Perceptions of Medicine in Pre-industrial Society*, ed. Roy Porter, Cambridge 2002; Roy Porter, ‘The Patient’s View: Doing Medical History from Below’, *Theory and Society* 14, 1985, pp. 175–98.

18 Handley, *Sleep*, pp. 31–8; William F. Maclehose, ‘Captivating Thoughts: Nocturnal Pollution, Imagination and the Sleeping Mind in the Twelfth and Thirteenth Centuries’, *Journal of Medieval History* 46: 1, 2020, pp. 98–131.

19 Werrett, *Thrifty Science*.

20 On early modern recipe collections see Leong, *Recipes and Everyday Knowledge*; Elaine Leong, ‘Making Medicines in the Early Modern Household’, *Bulletin of the History of Medicine* 82, 2008, pp. 145–68; Alisha Rankin, *Panacea’s Daughters*; Amanda Herbert, *Female Alliances: Gender, Identities, and Friendship in Early Modern Britain*, New Haven 2014, esp. pp. 78–116; *Reading and Writing Recipe Books*, ed. DiMeo and Pennell; Wendy Wall, *Recipes for Thought: Knowledge and Taste in the Early Modern Kitchen*, Philadelphia 2016; Anne Stobart, *Household Medicine in Seventeenth-Century England*, London 2016, esp. pp. 29–54.

21 Herbert, *Female Alliances*, p. 104.

22 Leong, *Recipes and Everyday Knowledge*, p. 3.

23 Wall, *Recipes for Thought*, p. 212. On the value of trustworthiness for recipes see Elaine Leong and Sara Pennell, ‘Recipe Collections and the Currency of Medical Knowledge in the Early Modern Marketplace’, in *Medicine and the Market in England and its Colonies c.1450–c.1850*, ed. Patrick Wallis and Mark Jenner, London 2004, pp. 133–52.

24 Some recipes would be collected on the off chance a member of the family required them; Leong, ‘Making Medicines’, p. 167.

25 Wall, *Recipes for Thought*, pp. 213–216. Sara Pennell, ‘Perfecting Practice? Women, Manuscript Recipes and Knowledge in Early Modern England’, in *Early Modern Women’s Manuscript Writing: Selected Papers from the Trinity/Trent Colloquium*, ed. Victoria Burke and Jonathan Gibson, Burlington 2004, pp. 237–58, esp. pp. 247–51; Leong, ‘Making Medicines’. On bio-chemical analysis of fingerprints on recipe books see Stefan Hanß, Samantha Greeves, Gleb Zilberstein, Julianne Simpson, Tony Richards, Svetlana Zilberstein, Pier Giorgio Righetti, Elisabeth Carr, ‘The Scientific Analysis of Renaissance Recipes: Proteomics, Medicine, and the Body in the Material Renaissance’, *American Historical Review* rhaf405, 2025, https://doi.org/10.1093/ahr/rhaf405.

26 On the value of trustworthiness see Elaine Leong and Sara Pennell, ‘Recipe Collections and the Currency of Medical Knowledge in the Early Modern Marketplace’, in Jenner and Wallis, *Medicine and the Market*, pp. 133–52. On the crossover between print and manuscript see Leong, *Recipes and Everyday Knowledge*, pp. 147–72.

27 Leong, *Recipes and Everyday Knowledge*, esp. pp. 147–172.

28 Leong, *Recipes and Everyday Knowledge*, p. 165.

29 Wendy Wall, ‘The World of Recipes: Intellectual Culture in and around the Seventeenth-Century Household’ in *The Oxford Handbook of Early Modern Women’s Writing in English, 1540–1700*, ed. Elizabeth Scott-Baumann, Danielle Clarke and Sarah C. E. Ross, Oxford 2022, pp. 319–34, esp. pp. 327–8; Julia Martins, ‘The Afterlife of Italian Secrets: Translating Medical Recipes in Early Modern Europe’ in *Crossing Borders, Crossing Cultures: Popular Print in Europe (1450*–*1900)*, ed. Massimo Rospocher, Jeroen Salman and Hannu Salmi, Berlin 2019, pp. 181–98.

30 Leong, *Recipes and Everyday Knowledge*, p. 14; Wall, *Recipes for Thought*, pp. xii, 6–7. On women’s involvement in recipe collection and creation see Herbert, *Female Alliances*, pp. 105–7; Rankin, *Panacea’s Daughters*, pp. 62–3.

31 Sasha Handley, ‘Sleep-Piety and Healthy Sleep in Early Modern English Households’, in *Conserving Health in Early Modern Culture*, ed. Sandra Cavallo and Tessa Storey, Manchester 2017, pp. 185–209, 200.

32 Herbert, *Female Alliances*, pp. 105–7.

33 Wall, *Recipes for Thought*, pp. xii, 6–7.

34 Rankin, *Panacea’s Daughters*, p. 62.

35 Michelle DiMeo, ‘‘‘Such a Sister Became Such a Brother”: Lady Ranelagh’s Influence on Robert Boyle,’ *Intellectual History Review* 25: 1, 2015, pp. 21–36; Michelle DiMeo, *Lady Ranelagh: The Incomparable Life of Robert Boyle’s Sister*, Chicago, 2024.

36 Leah Knight, *Of Books and Botany in Early Modern England: Sixteenth-Century Plants and Print Culture*, Burlington VT 2009.

37 Sarah Neville, *Early Modern Herbals and the Book Trade: English Stationers and the Commodification of Botany*, Cambridge 2022, pp. 19–22, 36.

38 Michael R. Best, ‘Medical Use of a Sixteenth-Century Herbal: Gervase Markham and the Bankes Herbal’, *Bulletin of the History of Medicine* 53, 1979, pp. 449–58.

39 Rebecca Laroche, *Medical Authority and Englishwomen’s Herbal Texts, 1550*–*1650*, London 2009, pp. 6, 13.

40 Neville, *Early Modern Herbals and the Book Trade*, pp. 36, 38. See also Sachiko Kusukawa, *Picturing the Book of Nature: Image, Text, and Argument in Sixteenth-Century Human Anatomy and Medical Botany*, Chicago 2012, p. 120.

41 John Parkinson, *Theatrum Botanicum: The Theater of Plants*, London 1640; John Gerard, *The Herball or Generall Historie of Plantes. Gathered by John Gerarde of London, Master in Chirurgerie, very much enlarged and amended by Thomas Johnson, citizen and apothecary of London*, London 1633; Nicholas Culpeper, *The English Physitian Enlarged*, London 1653; John Pechey, *The Compleat Herbal of Physical Plants*, London 1694. Parkinson’s herbal was the most comprehensive in scope, and contained thirty-two soporifics, followed by Gerard and Johnson (twenty-four), Turner (sixteen), Culpeper (sixteen), and Pechey (eleven). There was significant overlap between Parkinson’s herbal and that later published by Nicholas Culpeper, though Culpeper halved the number of soporifics in his shorter and more accessible volume, priced at just 3d. Graeme Tobyn, ‘Culpeper’s Herbal *The English Physitian* and its Debt to Apothecary John Parkinson’, *Medical History* 68: 3, 2024, pp. 237–53; Sarah Neville, *Early Modern Herbals and the Book Trade: English Stationers and the Commodification of Botany*, Cambridge 2021.

42 Laroche, *Medical Authority*, 17.

43 Tobyn, ‘Culpeper’s Herbal *The English Physitian*’, p. 240.

44 Parkinson, *Theatrum Botanicum*, pp. 1191–2; Gerard, *Herball*, p. 35.

45 Kusukawa, *Picturing the Book of Nature.*

46 Gerard, *Herball*, pp. 358–9.

47 Gerard, *Herball*, p. 359.

48 Parkinson, *Theatrum Botanicum*, p. 348.

49 Parkinson, *Theatrum Botanicum*, pp. 755–7.

50 Parkinson, *Theatrum Botanicum*, pp. 267–8.

51 Parkinson, *Theatrum Botanicum*, p. 1452.

52 Werrett, *Thrifty Science*, p. 11. Culpeper encouraged his readers to forage for medical materials and listed the places where key plants could be found in his *English Physitian.*

53 Culpeper, *English Physitian*, p. 107.

54 Pechey, *Compleat Herbal*, p. 159; Culpeper, *English Physitian*, p. 141.

55 Culpeper, *English Physitian*, p. 142.

56 Matthew Milner, *The Senses and the English Reformation*, Farnham 2011, p. 7.

57 Pavel Gregoric and Jakob Leth Fink, ‘Introduction: Sleeping and Dreaming in Aristotle and the Aristotelian Tradition’, in *Forms of Representation in the Aristotelian Tradition. Volume Two: Dreaming*, ed. Christina Thomsen Thörnqvist and Juhana Toivanen, Leiden 2022, pp. 1–27.

58 Francis Bacon, *Sylva Sylvarum*, ed. William Rawley, London 1628, second edition, p. 187.

59 ‘Commonplace book and recipes etc of Christopher Towneley (1604–74)’, A.2.121, Chetham’s Library, fols.12r–13r.

60 Doina-Cristina Rusu, ‘Animal Spirits: Bridging Mind and Body in Early Modern Philosophy’, in *Encyclopedia of Early Modern Philosophy and the Sciences*, ed. D. Jalobeanu and C. T. Wolfe, Cham 2021, pp. 1–5.

61 It was important that the extremities did not become too cold, however, since this would prevent sleep. See for instance Bacon, *Sylva Sylvarum*, p. 186.

62 Thomas Sheppey, ‘Book of choice receipts collected from several famous authors a great part in monasteries and often experimented as to a great number of them’, V.a.452, Folger Shakespeare Library (hereafter FSL), ca. 1675, p. 318. On the cold and dry nature of willow see Gerard, *Herball*, p. 1392.

63 See ‘Pharmaceutical and Cookery Recipes, ca. 1675’, V.a.21, FSL, 1675, p. 18; ‘The Lady Grace Castleton’s Book of receipts’, V.a.600, FSL, 17^th^ century, p. 123.

64 Both mallow and onion were said to cause sleepiness when consumed; see e.g. Gerard, *Herball*, pp. 171, 935.

65 ‘Receipt Book’, V.b.400, FSL, late 17^th^ – early 18^th^ century, p. 135. It is unclear exactly what ‘septialicum’ refers to in this recipe. It might relate to the Latin *septicus*, ‘putrefying’, suggesting it was aimed at treating putrefaction in the feet, or that the plaster itself would break down, causing the socks to fall off. Its precise definition is not required to appreciate the recipe’s focus on treating the body’s extremities with consequences for the brain and sleep.

66 ‘Medical recipes, compiled ca.1625’, X.d.469, FSL, 1625, p. 23.

67 As previous note, p. 46.

68 Handley, *Sleep*, pp. 22–9. Similar recipes in our sample include Richard Perssehouse, ‘Bifolium miscellany of medical, domestic and veterinary receipts’, X.d.738, FSL, ca.1643; ‘Cookery and medicinal recipes of the Granville family from Worcestershire and Cadiz’, V.a.430, FSL, 1640–1750, pp. 1, 90; ‘A Commonplace Book including Medical, Culinary, Arboricultural and Fishing Recipes, Charms and Instructions’, Harley MS 2389, British Library (hereafter BL), 16^th^ century, fol. 37v; ‘Receipt Book’, Harley MS 130, BL, 4^th^ quarter of the 16^th^ century, fol. 12r.

69 Parkinson, *Theatrum Botanicum*, p. 733.

70 Thomas Carter, ‘A booke of Phisicke, Surgery, Preserves and Cookery, with sundry other excellent Receipts’, MS.182, WL, fol. 27v. See also Sheppey, ‘Book of choice receipts’, V.a.452, p. 137.

71 Handley, *Sleep*, pp. 31–8.

72 William Tullett, *Smell in Eighteenth-Century England: A Social Sense*, Oxford 2019, p. 1.

73 Sandra Cavallo, ‘Health, Air and Material Culture in the Early Modern Italian Domestic Environment’, *Social History of Medicine* 29: 4, 2016, pp. 695–716, 709.

74 David Karmon and Christy Anderson, ‘Early Modern Spaces and Olfactory Traces’, in *The Routledge Handbook of Material Culture in Early Modern Europe*, London 2016, p. 357.

75 Elizabeth Grey, *A Choice Manual, or Rare Secrets in Physick and Chirurgery*, London 1687, p. 71.

76 See Gerard, *Herball*, pp. 681, 1263, 1536.

77 Henry Pagett, ‘Cookbook, ca. 1678–ca. 1689’, V.b.13, FSL, 1678–1689, fol. 6 r.

78 Tullett, *Smell*, pp.27–28. On bad smells dulling the spirits see Ambroise Paré, *The workes of that famous chirurgion Ambrose Parey translated out of Latine and compared with the French*, London 1634, p. 823.

79 Handley, *Sleep*, p. 33.

80 John Floyer, *The Touch-Stone of Medicines*, London, 1687, p. 169.

81 See for example, ‘Pharmaceutical recipes’, V.b.286, FSL, 1690–1870, p. 22.

82 See John de Feckenham, ‘This book of sovereign medicines against the most common and known diseases … collected of Master Doctor Feckenham, late Abbot of Westminster [manuscript], ca. 1600’, V.b.129, FSL, 1600, fol. 69 v; Henry Dineley, ‘Collection of medical receipts’, MS.244, WL, p. 273; Charles Leighton, ‘Esculapius redivivus; a notable treasury of health and wisdom’, MS.3229, WL, p. 21; Anonymous, ‘A Booke of excellent medicines’, MS.142, WL, mid 17^th^ century, fol. 17v; Nicholas Culpeper, *Culpeper’s School of* Physick, London, 1659, p. 103.

83 Sheppey, ‘Book of Choice Receipts’, V.a.452, FSL, p. 321.

84 Leighton, ‘Esculapius redivivus’, MS.3229, WL, p. 21.

85 Feckenham, ‘Book of sovereign medicines’, V.b.129, FSL, fol. 69v.

86 Alexander Ross, *Arcana microcosmi*, London 1652, p. 102.

87 ‘Receipt Book’, V.b.400, FSL, p. 134.

88 ‘Pharmaceutical recipes’, V.b.286, FSL, p. 53.

89 ‘Pharmaceutical and cookery recipes’, V.a.21, FSL, p. 18. This recipe also appears in ‘A Booke of excellent medicines’, MS.142, WL, mid 17th century, fol. 17v.

90 On the binding qualities of ‘lees of wine’ see Gerard, *Herball*, p. 883. Gerard noted that wine itself encouraged sleep when consumed.

91 On civet see Sarah-Maria Schober, ‘Taming the Untamable: Early Modern Civet Cats and the Nature-Culture Dichotomy’, in *Humans and Other Animals*, ed. Joanna Woodall, Eric Jorink, Edward Wouk, Leiden 2021, pp. 32–57; Harald H. Roth and Günter Merz, ‘Wildlife Products’, in *Wildlife Resources: A Global Account of Economic Use*, ed. Harald H. Roth and Günter Merz, Berlin 2013, pp. 331–86, 383.

92 Cavallo, ‘Health, Air and Material Culture’, pp. 712–13.

93 As previous reference.

94 Evelyn Welch, ‘Scented Buttons and Perfumed Gloves: Smelling Things in Renaissance Italy’, in *Ornamentalism: The Art of Renaissance Accessories*, ed. Bella Mirabella, Ann Arbor 2011, pp. 13–39.

95 See for example Thomas Tryon, *A treatise of cleanness in meats and drinks of the preparation of food, the excellency of good airs and the benefits of clean sweet beds also of the generation of bugs and their cure*, London 1682, p. 7; John Trusler, *An Easy Way to Prolong Life, by a little attention to what we eat and drink*, London 1775, p. 16.

96 Quoted in Handley, *Sleep*, p. 48.

97 ‘Mary Doggett Book of Receipts’, Add MS 27466, British Library, 1682, pp. 32–3; Recipe book of Lady Ann Fanshawe (1625–1680), MS.7113, WL, 1651–1707, pp. 194, 198–9.

98 Recipe book of Ann Charlotte Frescheville, Folio 164 74x098, Winterthur Library, 1669, pp. 62, 84; Recipe book of Lady Frances Catchmay, MS.184a, WL, 1625, p. 2; Recipe book of Lady Catherine Fitzgerald, MS.2367, WL, p. 76; Recipe book of Lady Barrett, MS.1071, WL, pp. 348–50.

99 Cavallo, ‘Health, Air and Material Culture’, pp. 709–10.

100 Jodocus Lommius, *A Treatise of Continual Fevers…To which are added, Medical observations*, trans. Thomas Dale, London 1732, p. 112.

101 Recipe book of Ann Charlotte Frescheville, Folio 164 74x098, Winterthur Library, p. 84.

102 On the cold nature of benzoin see Nicholas Culpeper, *Pharmacopoeia Londinensis or the London Dispensatory. Further adorned by the Studies and Collections of the Fellows, now living of the said Colledg*, London 1653, p. 48. For its relation to the chest and head see George Wilson, *A complete course of chymistry*, fifth edition, London 1736, pp. 296–7.

103 See Nigel Groom, *New Perfume Handbook*, second edition, London 1997, p. 32.

104 Gerard, *Herball*, p. 1263.

105 John Partridge, *The treasurie of commodious conceits, & hidden secrets and may be called, the huswiues closet, of healthfull prouision*, London 1573, p. 18.

106 Gerard, *Herball*, p. 1400.

107 Handley, *Sleep*, p. 46.

108 See for example Humphrey Brooke, *Ugieine or A conservatory of health*, London 1650, p. 70.

109 Recipe book of Mrs. Carr, MS.1511, WL, 1682, fol. 57 v.

110 Sheppey, ‘Book of Choice Receipts’, V.a.452, FSL, fol.318.

111 Pechey, *Compleat Herbal*, p. 71.

112 Handley, *Sleep*, p. 65. A recipe for cushions filled with roses appears in ‘A Large collection of quaint medical and other receipts in MSS’, MS.400, WL, c.1625–c.1725, pp. 19–20.

113 Partridge, *The Treasurie*, p. 18.

114 ‘English Recipe Book, 17^th^ century –18^th^ century’, MS.8097, WL, 17^th^–18^th^ century, p. 109.

115 Other examples appear in ‘English cookery and medicine book’, V.b.380, FSL, 1677–1711, p. 249; Recipe book of Lady Ann Fanshawe, MS.7113, WL, p. 198; ‘Recipes of the Maunsell & Caswell families’, MS 51,541/3, National Library of Ireland, 1732–1860, n.p.

116 Jacqui Carey, *Sweet Bags: An Investigation into 16^th^ and 17^th^ Century Needlework*, Ottery St Mary 2009.

117 ‘English Recipe Book’, MS.8097, WL, p. 108.

118 Gerard, *Herball*, p. 1558; William Coles, *Adam in Eden, or nature’s paradise*, London 1657, p. 31.

119 Francis Steer, ‘The Inventory of Arthur Coke of Bramfield, 1629’, *Proceedings of the Suffolk Institute of Archaeology* 25: 3, 1951, pp. 264–87, 280. Cited in Carey, *Sweet Bags*, p. 9.

120 John Evelyn, *Fumigugium: Or the Inconvenience of the Aer and Smoak of London Dissipated*, London, 1661, pp. 14–15.

121 Handley, *Sleep*, p. 43. On health and air quality see also Alexandra Logue, ‘“Saucy Stink”: Smells, Sanitation and Conflict in Early Modern London’, *Renaissance and Reformation* 44: 2, 2021, pp. 61–86.

122 Recipe book of Lady Frances Catchmay, MS.184a, WL, p. 2.

123 See Handley, *Sleep*, pp. 6–7. On the connection of vulnerability to material practices of sleep see p. 51.

124 ‘Orvietan: A Counter-Poyson against the Infectious Ayr of London’, Sloane MS 621, BL, p. 85; Gerard, *Herball*, p. 1373.

125 ‘Orvietan’, Sloane MS 621, BL, p. 86.

126 Evelyn, *Fumigugium*, pp. 24, 1.

127 William Cavert, *The Smoke of London: Energy and Environment in the Early Modern City*, Cambridge 2016, p. 174.

128 See A. Roger Ekirch, ‘Sleep We Have Lost: Pre-Industrial Slumber in the British Isles’, *The American Historical Review* 106, 2001, pp. 343–86, 348. See also A. Roger Ekirch, *At Day’s Close: A History of Nighttime*, London 2005; A. Roger Ekirch, ‘The Modernization of Western Sleep: Or, Does Insomnia Have a History?’, *Past and Present* 226, 2015, pp. 149–92. Ekirch’s arguments regarding biphasic sleep have recently been contested in Niall Boyce, ‘Have We Lost Sleep? A Reconsideration of Segmented Sleep in Early Modern England’, *Medical History* 67, 2023, pp. 91–108.

129 Rublack, ‘Matter in the Material Renaissance’, p. 46.

## Author Biographies

**Holly Fletcher** is a Wellcome Trust Early Career Fellow at University College London. Her research focuses on the history of the body, material culture, and environmental history. Fletcher has published several prize-winning articles in leading journals such as *Historical Research*, *German History*, and *Gender & History*, and her monograph *Body Size in Early Modern Germany* is forthcoming with Oxford University Press. Fletcher is PI of the Wellcome-funded project ‘The Fats of Life in the Early Modern World, 1500–1700: Matter in Multispecies Medicine’ (2025–30).

**Sasha Handley** is Professor of Early Modern History at the University of Manchester. Her work focuses on material culture, healthcare practices, and environmental history. Handley’s monographs include *Sleep in Early Modern England* (2016, winner of the Social History Book Prize 2017) and *Visions of an Unseen World: Ghost Beliefs and Ghost Stories in Eighteenth-Century England* (2007). In 2023–24 she co-curated the major exhibition *Albrecht Dürer’s Material World* at The Whitworth. Handley is the PI of the Wellcome Trust funded project ‘Sleeping well in the early modern world: an environmental approach to the history of “sleep care”’ (2021–26).

## NOTES AND REFERENCES

The research for this article was undertaken as part of the Wellcome Trust funded project ‘Sleeping Well in the Early Modern World: An Environmental Approach to the History of Sleep Care’. We would like to thank Dr Leah Astbury and Dr Abigail Greenall for their contributions to this project which informed the development of this article. We also thank Professor Stefan Hanß for reading an earlier draft of the text.

